# Schrenk spruce leaf litter decomposition varies with snow depth in the Tianshan Mountains

**DOI:** 10.1038/s41598-020-76368-9

**Published:** 2020-11-11

**Authors:** Lu Gong, Xin Chen, Xueni Zhang, Xiaodong Yang, Yanjiang Cai

**Affiliations:** 1 College of Resources and Environment Science, Xinjiang University, Urumqi, 830046 China; 2 Key Laboratory of Oasis Ecology, Ministry of Education, Urumqi, 830046 China; 3 State Key Laboratory of Subtropical Silviculture, Zhejiang A&F University, 666 Wusu Street, Lin’an District, Hangzhou, 311300 China

**Keywords:** Biogeochemistry, Forest ecology, Biogeochemistry, Ecology

## Abstract

Seasonal snowfall, a sensitive climate factor and the main form of precipitation in arid areas, is important for forest material circulation and surface processes and profoundly impacts litter decomposition and element turnover. However, how the thickness and duration of snow cover affect litter decomposition and element release remain unclear. Thus, to understand the effects of snow on litter decomposition, fiber degradation and their relationships with soil properties, a field litterbag experiment was conducted under no, thin, medium, and thick snow cover in a Schrenk spruce (*Picea schrenkiana*) forest gap in the Tianshan Mountains. The snow cover period exhibited markedly lower rates of decomposition than the snow-free period. The litter lignin, cellulose and N concentrations in the pregrowing season and middle growing season were significantly higher than those in the deep-freeze period, and the litter C and P concentrations were significantly higher during the onset of the freeze–thaw period, deep-freeze period and thaw period than in the late growing season. The litter cellulose, C and N concentrations were significantly higher under thick snow cover than under no snow cover in most stages. Moreover, the correlations among litter mass, cellulose, lignin/cellulose and soil bulk density varied with snow cover depth. The temporal variations and snow cover depth affected the decomposition process significantly. The former affected lignin, cellulose and P, and the latter affected cellulose, C and N and changed the litter-soil properties relationship. These differences provide references for understanding how winter conditions affect material cycling and other ecological processes under climate change.

## Introduction

Litter decomposition is a key process of material cycling and energy flow in forest ecosystems, especially for maintaining ecological functions ^1–3^. As the main method of returning carbon (C) and nutrients in forests, litter decomposition transports organic matter and nutrient elements to soil to maintain soil fertility, promotes plant growth and development^4–6^, provides energy sources for soil microorganisms and small animals, and facilitates various ecological processes, such as soil maturation and soil organic matter turnover^7,8^. Litter decomposition is thus the basis for exploring C turnover and nutrient cycling in various forest ecosystems, and it has been an important focus in the study of biogeochemical cycling in terrestrial ecosystems ^5,9,10^.

Litter decomposition is a complex process controlled by both abiotic and biotic factors. Among these factors, climatic factors can affect litter matrix quality and change microbial metabolism and activity, thus playing a critical role in regulating litter decomposition ^11–13^. Traditional ecological theory holds that litter decomposition increases with temperature and humidity but would be slow or even stagnant at low temperature in winter ^14^. However, an increasing number of studies have shown that litter decomposition in winter plays an important role in the interannual dynamics of material and nutrition circulations at middle to high latitudes ^15–17^. Seasonal snow cover at the landscape scale is an important factor that controls the structure and function of mountain forest ecosystems, and it provides an adiabatic and relatively stable environment for litter decomposition. In winter, snow patches with different depths and durations from the middle of a gap to the understory can be formed in forest ecosystems due to the effects of canopy shelter, flow collection and wind, which are accompanied by different freeze–thaw patterns that profoundly affect litter decomposition ^18^. At present, warm winters and extreme climatic events caused by global climate change are changing the pattern of winter snow cover ^19^. Uncovering the effect of different snow cover depth conditions on litter decomposition in mountain forest ecosystems is helpful for exploring the regulatory mechanisms of litter decomposition under seasonal snow cover. Moreover, it is of great scientific significance to understand the dynamics of material cycling and energy conversion in winter forest ecosystems under climate warming and to accurately evaluate forest ecology.

Bratt et al. ^20^ pointed out that litter decomposition in winter accounted for 26% of the annual decomposition, while Wu et al. ^21^ found a contribution of 42.5–65.5%. These results indicated that winter forest litter decomposition is vital to the entire decomposition process, and differences in snow cover may significantly impact litter decomposition. Thick snow cover has been found to promote litter decomposition in a middle- to high-latitude mountain forest ecosystem in northeastern North America^22^, and greater litter decomposition rates in coniferous forests has been reported under thick than under thin snow cover ^18,23^. The loss and decomposition rates of lignin and cellulose under thick snow cover have also been found to be higher than those under thin or no snow cover ^24,25^. The substrate quality, such as chemical composition, humification process and enrichment/release of elements of litter under snow, may therefore vary depending on the snow cover depth ^26^. Additionally, the pattern of snow patches in winter not only affects the forest litter decomposition during the snow cover period but also continues to influence the litter decomposition in the following growth season ^11,17,27^. Nevertheless, few studies have focused on the response of litter decomposition to snow cover in arid mountainous areas.

As an important component of the mountainous oasis basin system in the arid region of western China, the Tianshan Mountains is also the largest mountain system in central Asia. Schrenk spruce (*Picea schrenkiana*), an endemic species of central Asia, is the dominant tree species in the Tianshan Mountains ^28^. As mentioned above, winter forest litter decomposition plays an important role in decomposing litter, and the decomposition may vary with snow cover depth, however, few studies have investigated the decomposition of Schrenk spruce forest litter in the Tianshan Mountains. Seasonal snow cover is the most active and sensitive environmental factor in this region, and the snow cover usually lasts for 5–6 months ^29^. Furthermore, snowfall and the seasonal freeze–thaw cycle can produce a natural snow cover gradient associated with forest gaps ^29,30^. To date, the following questions remain unanswered: what are the characteristics of litter decomposition and the regularity of C, nitrogen (N), and phosphorus (P) release in Schrenk spruce forests during the snow cover period in this area? Does the depth of snow cover significantly affect the process of litter decomposition? Therefore, the characteristics of litter decomposition and the dynamics of matrix quality in Schrenk spruce forests under snow cover with different depths were investigated in this study. We then hypothesized that (1) the difference in litter decomposition and element release characteristics vary in different decomposition stages, (2) thicker snow cover could promote the decomposition of litter and accelerate the release of C and nutrients from litter, and (3) thicker snow cover could strengthen the relationship between litter and soil physical and chemical properties. Therefore, the major purpose of this study was to reveal whether leaf litter decomposition varies with snow depth and identify the driving mechanisms in mountain forest ecosystems of arid areas. Moreover, this study provides small-scale data for further understanding the impact of winter environmental changes on material cycling and other ecological processes under climate change.

## Materials and methods

### Site description

The experiment was conducted at a site adjacent to the Astronomical Observatory of Nanshan in the northern foothills of the Tianshan Mountains, Xinjiang, China (43°29′37″ N, 87.11°11′31″ E, 1930 m above sea level). The region has a temperate continental climate, with a mean annual temperature of 2.8 ºC and an average annual precipitation of 176 mm. The snow cover generally lasts for 5–6 months (from November to the following April), with a maximum snow depth of more than 100 cm. This region is characterized by needleleaf forest, which is dominated by Schrenk spruce. The soil is classified as Folic Cambisols (Loamic, Humic) according to the WRB (World Reference Base for Soil Resources) classification.

### Experimental design and field sampling

**Figure 1:**
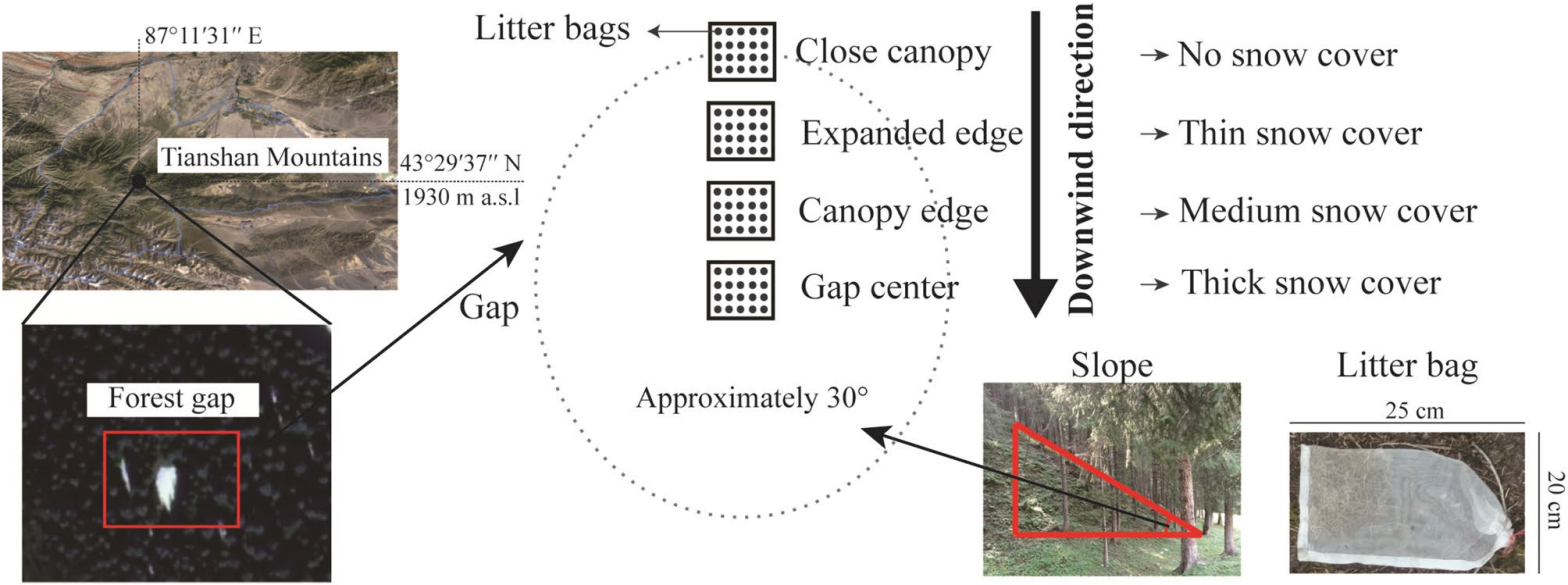
Schematic map of the experimental design.

Freshly fallen leaf litter was collected from Schrenk spruce in mid-September 2017. All litter samples were air dried at room temperature for two weeks, and then 10 g of each sample was put in a 20 cm × 25 cm litterbag with a mesh size of 1.0 mm on the top and 0.5 mm on the bottom. Three forest gaps with similar topographical factors (such as elevation, slope and wind direction) were selected as sample areas. The forest gaps were at least 400 m^2^ in size and separated by at least 1 km. Four sample plots per gap were arranged in a line from the center of the forest gap to its edge (under a closed canopy), which ran parallel to the main wind direction. These four sample plots represented four natural snow cover depths (thick, medium, thin and no snow cover), and the distance between the plots was roughly 5 m Fig. 1.

All litterbags were randomly assigned to sample plots upon the soil surface on October 14, 2017, and the litterbags were fixed to the soil surface with a small bamboo stick. After the litterbags were placed, sampling was conducted every two months. The experimental year was then divided into six stages, i.e., the onset of the freeze–thaw period (FTP, December 2017), deep-freeze period (DFP, February 2018), thaw period (TP, April 2018), pregrowing season (PGS, June 2018), middle of the growing season (MGS, August 2018), and late growing season (LGS, October 2018). Three litterbags per snow cover depth condition were collected from each plot at the end of each stage. The topsoil (0–10 cm) under the litterbags was collected synchronously at each sampling time. A total of 216 litterbags (3 gaps × 4 snow cover depths × 6 stages × 3 replicates) were retrieved in this study.

### Analytical methods and calculations

The snow depth was obtained by averaging the direct measurements from five random locations with a ruler for each snow cover depth condition after snowfall events (Table 1). iButton DS1923-F5 recorders (Maxim Integrated Products, Inc., Sunnyvale, TX, USA) were used to record the land surface temperature at the litter layer for each snow cover depth (Fig. 1). The litter remaining in each litterbag after collection was manually separated from foreign materials, oven dried at 65 °C and weighed to determine the dry mass loss. The oven-dried litter samples were then ground to a size of < 1 mm^31^. Soil samples from each plot were air-dried and ground to a size of 0.15 mm. The C, N and P concentrations were measured for both the litter and soil samples. The total C concentration was determined using the dichromate oxidation-ferrous sulfate titration method. The total N concentration was determined using Kjeldahl digestion. The total P concentration was measured using the molybdenum-blue colorimetric method. The lignin and cellulose concentrations were determined with the acid detergent lignin method^25^. The soil pH and electrical conductivity were measured using potentiometry and the conductivity method, respectively (at a soil:water ratio of 1:5). The soil water content was determined by the oven-dried method. The soil bulk density was determined using the standard cutting ring with a volume of 100 cm^3^ and oven drying to constant weight at 105 °C.

**Table 1:** The depth of seasonal snow cover at different stages. Different lowercase letters in the same snow cover indicate a significant difference among different stages (*P* < 0.05).

Period	Stage	No snow cover (cm)	Thin snow cover (cm)	Medium snow cover (cm)	Thick snow cover (cm)
Snow cover period	FTP	–	16.02 ± 3.80 b	24.66 ± 3.71 b	36.03 ± 3.56 b
	DFP	–	23.13 ± 5.00 a	31.59 ± 4.58 a	46.27 ± 5.31 a
	TP	–	5.07 ± 1.53 c	6.13 ± 2.88 c	8.67 ± 1.58 c
Snow-free period	PGS	–	–	–	–
	MGS	–	–	–	–
	LGS	–	–	–	–

The dry mass loss (L, %) and the element, lignin and cellulose release (R, % of initial dry mass) of the leaf litter were calculated as follows:

$$L_{t} = {{\left( {M_{{t{ - }1}} { - }M_{t} } \right)} \mathord{\left/ {\vphantom {{\left( {M_{{t{ - }1}} { - }M_{t} } \right)} {M_{0} }}} \right. \kern-\nulldelimiterspace} {M_{0} }} \times 100{{\% }}$$

$$R(\% ) = {{(M_{0} C_{0} - M_{t} C_{t} )} \mathord{\left/ {\vphantom {{(M_{0} C_{0} - M_{t} C_{t} )} {M_{0} }}} \right. \kern-\nulldelimiterspace} {M_{0} }}C_{0} \times 100\%$$

where L_t_ (%) is the mass loss at time t; M_t−1_ (g) and M_t_ (g) are the dry masses of remaining litter at time t − 1 and t, respectively; M_0_ (g) is the initial dry mass of litter; C_0_ (g/kg) is the initial concentration of element, lignin and cellulose in litter; and C_t_ (g/kg) is the concentration of element, lignin and cellulose at sampling time t. The leaf litter decomposition constant (*k*) for the entire year of the experiment was calculated using a single exponential model^32^:

$${{M_{t} } \mathord{\left/ {\vphantom {{M_{t} } {M_{0} }}} \right. \kern-\nulldelimiterspace} {M_{0} }} = a{\text{e}}^{{\text{ - kt}}}$$

$$T_{0.50} = - {{\ln \left( {1 - 0.50} \right)} \mathord{\left/ {\vphantom {{\ln \left( {1 - 0.50} \right)} k}} \right. \kern-\nulldelimiterspace} k}$$

$$T_{0.95} = - {{\ln \left( {1 - 0.95} \right)} \mathord{\left/ {\vphantom {{\ln \left( {1 - 0.95} \right)} k}} \right. \kern-\nulldelimiterspace} k}$$

where *a* is the fitting parameter; *k* is the annual decomposition rate of litter; T_0.50_ (a) is the time to 50% decomposition of litter; and T_0.95_ (a) is the time to 95% decomposition of litter.

### Statistical analysis

One-way analyses of variance (ANOVA) and Tukey’s post hoc multiple comparison test were used to compare the litter lignin, cellulose, C, N and P concentrations among different snow depth conditions and stages, and the level of significance was *P* = 0.05. Repeated measures ANOVA was used to analyze the effect of decomposition period and snow cover depth on the litter properties. If Mauchly’s test of sphericity was not significant (*P* < 0.05), the Greenhouse–Geisser method is used for correction (Supplementary Table S3). A redundancy analysis (RDA) and Pearson correlation coefficients were used to reveal the relationships and significance levels of physicochemical characteristics between the leaf litter and soil, and all data were Min–Max normalized before performing the RDA. All analyses were conducted in the statistical software SPSS (version 19.0), Excel (2010) or Canoco 4.5 for Windows.

## Results

### The classical statistical characteristics of soil and litter properties

The average soil pH, conductivity, soil water content, bulk density, C, N and P were 6.90, 0.34 mS/cm, 27.86%, 0.89 g/cm^3^, 129.78 g/kg, 1.24 g/kg and 0.52 g/kg, respectively (Table 2). The soil water content, bulk density, C, N and P under thick snow cover were higher than under no snow cover (Supplementary Table S1). The average litter dry mass, lignin, cellulose, C, N and P were 8.34 g, 284.74 g/kg, 169.25 g/kg, 463.28 g/kg, 10.36, and 1.26 g/kg, respectively (Table 2). Cellulose had the highest coefficient of variation (23.51%), followed by lignin (22.45%).

**Table 2:** The classical statistical characteristics of soil and litter properties.

Properties	Min	Max	Mean	Standard deviation	Skewness	Kurtosis	Coefficient of variation
**Soil**							
pH	6.01	7.65	6.90	0.42	− 0.37	− 0.64	6.05
Conductivity (mS/cm)	0.20	0.69	0.34	0.08	1.02	4.29	23.25
Water content (%)	19.29	39.97	27.86	5.19	0.42	− 0.52	18.64
Bulk density (g/cm^3^)	0.74	1.31	0.89	0.13	1.21	1.12	14.20
C (g/kg)	101.08	154.50	129.78	10.48	0.07	− 0.04	8.07
N (g/kg)	0.93	1.50	1.24	0.12	0.07	0.00	9.71
P (g/kg)	0.39	0.65	0.52	0.05	− 0.21	0.04	9.78
C/N	96.67	115.76	105.10	5.60	0.15	− 1.33	5.33
C/P	193.27	384.99	250.55	33.67	1.72	4.68	13.44
N/P	1.77	3.66	2.39	0.34	1.34	3.26	14.17
**Litter**							
Dry mass (g)	6.98	9.38	8.34	0.71	− 0.19	− 1.35	8.50
Lignin (g/kg)	175.17	417.37	284.74	63.92	0.18	− 0.87	22.45
Cellulose (g/kg)	98.10	272.47	169.25	39.79	0.75	0.28	23.51
C (g/kg)	422.55	509.02	463.28	20.40	0.20	− 0.43	4.40
N (g/kg)	6.54	13.47	10.36	1.57	− 0.31	− 0.71	15.14
P (g/kg)	1.01	1.53	1.26	0.11	0.03	− 0.38	8.63
C/N	31.37	74.23	45.96	8.43	0.73	0.39	18.33
C/P	311.70	446.41	369.60	29.95	0.52	0.10	8.10
N/P	4.80	11.38	8.32	1.69	− 0.08	− 0.92	20.28
C/lignin	1.03	2.84	1.72	0.44	0.60	− 0.36	25.53
C/cellulose	1.71	4.46	2.87	0.61	0.23	− 0.23	21.29
Lignin/cellulose	0.96	3.65	1.78	0.63	0.99	0.42	35.46

### Variation in snow cover depth and land surface temperature

**Figure 2:**
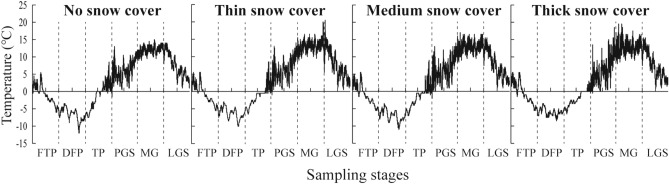
Dynamics of land surface temperature under different snow cover depths at different stages. FTP: freeze–thaw period; DFP: deep-freeze period; TP: thaw period; PGS: pregrowing season; MGS: middle of the growing season; LGS: late growing season.

The maximum snow depth for each snow cover condition appeared in the deep-freeze period (Table 1). The variation in land surface temperature under the four snow cover depth conditions was nearly the same, with a trend of decreasing, increasing and then decreasing. The lowest temperature (− 12.0 °C) and the highest temperature (20.5 °C) appeared in the deep-freeze period of the no snow cover and the middle of the growing season of the thick snow cover, respectively (Fig. 2).

### Mass loss and decomposition coefficient of litter

**Figure 3:**
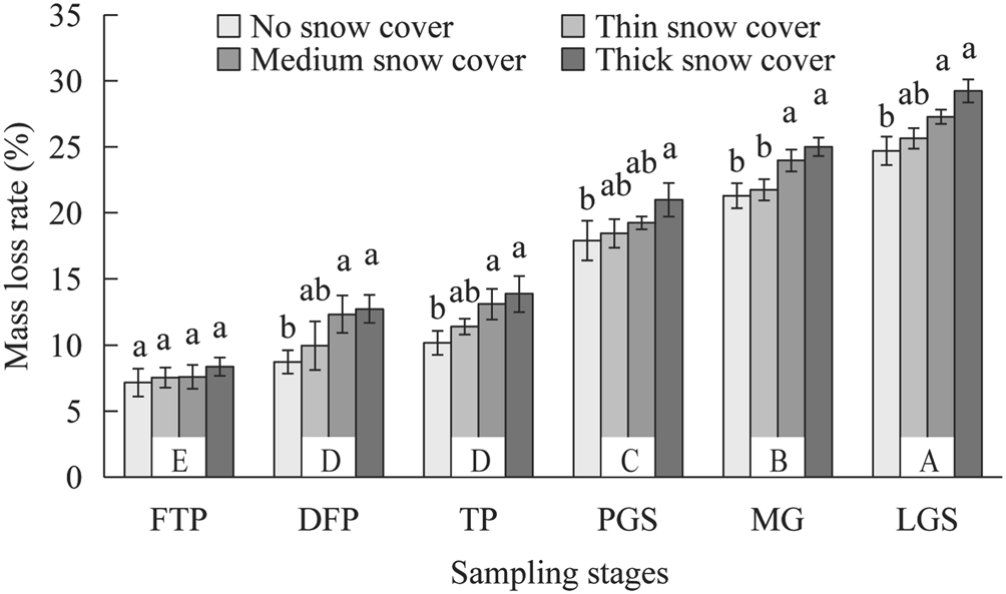
Dry mass loss of leaf litter under different snow cover depths at different stages. FTP: freeze–thaw period; DFP: deep-freeze period; TP: thaw period; PGS: pregrowing season; MGS: middle of the growing season; LGS: Late growing season. Different lowercase letters for the same stage indicate a significant difference among different snow cover depths (*P* < 0.05), and different uppercase letters indicate a significant difference among different stages (*P* < 0.05).

The dry mass loss of leaf litter increased with decomposition time, and differences were observed among the snow cover depth conditions (Fig. 3). The loss varied at different decomposition stages. Specifically, the loss in the late growing season was higher than other stages (*P* < 0.05), and the loss was significantly higher in the snow-free period (LGS, MGS and PGS) than in the snow cover period (TP, DFP and FTP). The loss under thick snow cover was significantly higher than that under no snow cover from the deep-freeze period to the late growing season. Based on Olson's exponential decay model, the decomposition coefficient (*k*) ranged from 0.277 to 0.322, and it increased sequentially with increasing snow cover depth (Table 3). No snow cover exhibited the longest half-decomposition time, while thick snow cover exhibited the shortest one.

**Table 3:** Decomposition characteristics of leaf litter based on Olson’s exponential decay model.

Snow cover depth	Regression model	Decomposition constant k	Coefficient (R^2^)	Time to 50% decomposition (a)	Time to 95% decomposition (a)
No	$y = 1.003e^{ - 0.277x}$	0.277	0.942	2.502	10.815
Thin	$y = 0.995e^{ - 0.280x}$	0.280	0.958	2.476	10.700
Medium	$y = 0.989e^{ - 0.299x}$	0.299	0.972	2.318	10.020
Thick	$y = 0.989e^{ - 0.322x}$	0.322	0.972	2.153	9.304
Entirety	$y = 0.994e^{ - 0.294x}$	0.294	0.964	2.358	10.190

### Dynamic characteristics and differences in lignin and cellulose in litter

**Figure 4:**
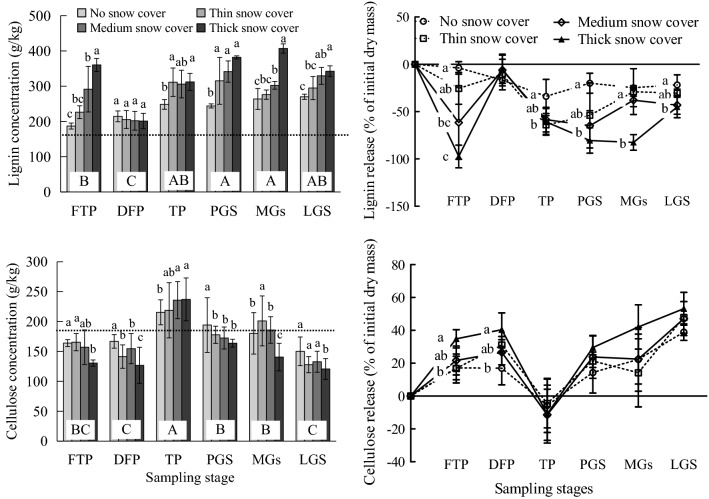
Effects of snow cover depth on the lignin and cellulose concentrations and release of leaf litter. The dotted line indicates the initial concentration. Different lowercase letters for the same stage indicate a significant difference among different snow cover depths (*P* < 0.05), and different uppercase letters for the same element indicate a significant difference among different stages (*P* < 0.05). FTP: freeze–thaw period; DFP: deep-freeze period; TP: thaw period; PGS: pregrowing season; MGS: middle of the growing season; LGS: late growing season.

The lignin concentration was significantly higher in the pregrowing season and middle of the growing season than in the freeze–thaw period and deep-freeze period (Fig. 4), and lignin release was negative. The cellulose concentration was higher in the thaw period than the pregrowing season and middle of the growing season (*P* < 0.05). The lignin concentration was significantly higher under the thick snow cover than no snow cover, and the lignin release under thick snow cover was significantly lower than that under no snow cover. Both the decomposition stage and snow cover depth (*P* < 0.01) affected litter lignin significantly (Table 4). The cellulose concentration under no snow cover was higher than that under thick snow cover in the freeze–thaw period, deep-freeze period, pregrowing season, middle of the growing season and late growing season (*P* < 0.05) but lower than that under thick snow cover in the thaw period (*P* < 0.05). The decomposition stage (*P* < 0.01) affected litter cellulose significantly (Table 4).

**Table 4:** Repeated measures ANOVA of litter properties.

Property	Variation source	df	F	Sig
Lignin	Snow cover depth (between-subjects)	3	36.352	< 0.001
	Decomposition stage (within-subjects)	2.607	58.145	< 0.001
	Depth × stage	7.822	4.846	0.002
Cellulose	Snow cover depth (between-subjects)	3	3.622	0.065
	Decomposition stage (within-subjects)	3.110	18.230	< 0.001
	Depth × stage	9.330	0.844	0.587
C	Snow cover depth (between-subjects)	3	25.886	< 0.001
	Decomposition stage (within-subjects)	6	36.571	< 0.001
	Depth × stage	18	0.923	0.557
N	Snow cover depth (between-subjects)	3	6.829	0.013
	Decomposition stage (within-subjects)	2.874	69.385	< 0.001
	Depth × stage	8.623	1.928	0.092
P	Snow cover depth (between-subjects)	3	2.970	0.097
	Decomposition stage (within-subjects)	3.044	17.682	< 0.001
	Depth × stage	9.133	1.070	0.418

### Litter C, N and P concentrations and their releases

**Figure 5:**
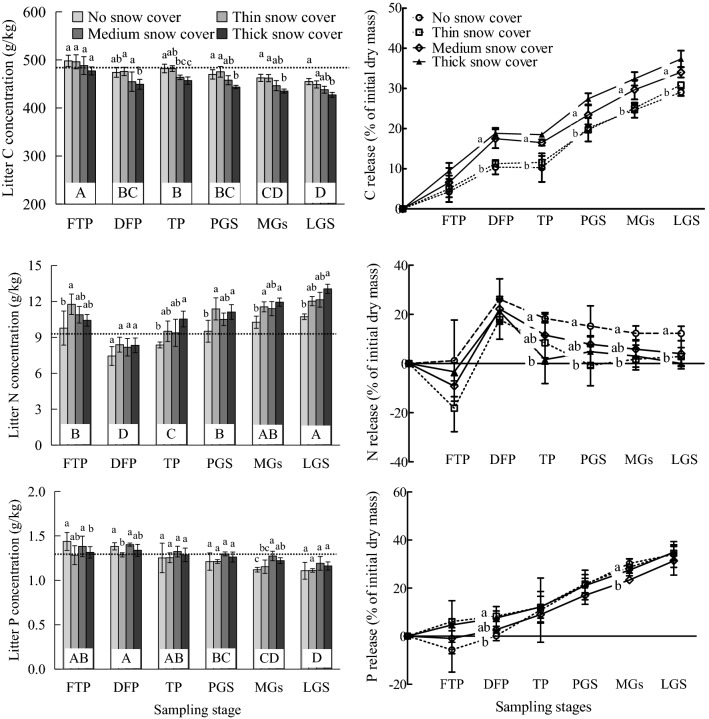
Effects of snow cover depth on the C, N and P concentrations and release of leaf litter. The dotted line indicates the initial concentration. Different lowercase letters for the same stage indicate a significant difference among different snow cover depths (*P* < 0.05), and different uppercase letters for the same element indicate a significant difference among different stages (*P* < 0.05). FTP: freeze–thaw period; DFP: deep-freeze period; TP: thaw period; PGS: pregrowing season; MGS: middle of the growing season; LGS: late growing season.

The litter C concentration was significantly higher in the freeze–thaw period, deep-freeze period and thaw period than in the late growing season, and the C release was gradually decreased (Fig. 5). The litter N concentration was significantly higher in the late growing season than the other stages, and it was significantly lower in the deep-freeze period than the other stages. The litter P concentration was higher (*P* < 0.05) in the freeze–thaw period, deep-freeze period and thaw period than the middle of the growing season and late growing season, and it was generally greater (*P* < 0.05) during the snow cover period than the snow-free period.

The litter C concentration was significantly lower under thick snow cover than for the other snow cover depths in all stages, and the release under thick snow cover was significantly higher than that of no snow cover in the thaw period, pregrowing season, middle of the growing season and late growing season. The litter N concentration under no snow cover was significantly lower than that under thick snow cover in the thaw period, pregrowing season, middle of the growing season and late growing season, while the litter N release under no snow cover was higher than that under thick snow cover in these stages (*P* < 0.05). The litter P concentration under thick snow cover was higher than that under no snow cover in middle of the growing season (*P* < 0.05), and there were no significant differences between the different snow cover depths in most stages. In general, both the decomposition stage and snow cover depth (*P* < 0.01) affected litter C significantly, and decomposition stage (*P* < 0.01) affected litter N and P significantly (Fig. 5 and Table 4).

### Relationship between litter decomposition and soil environmental factors

**Figure 6:**
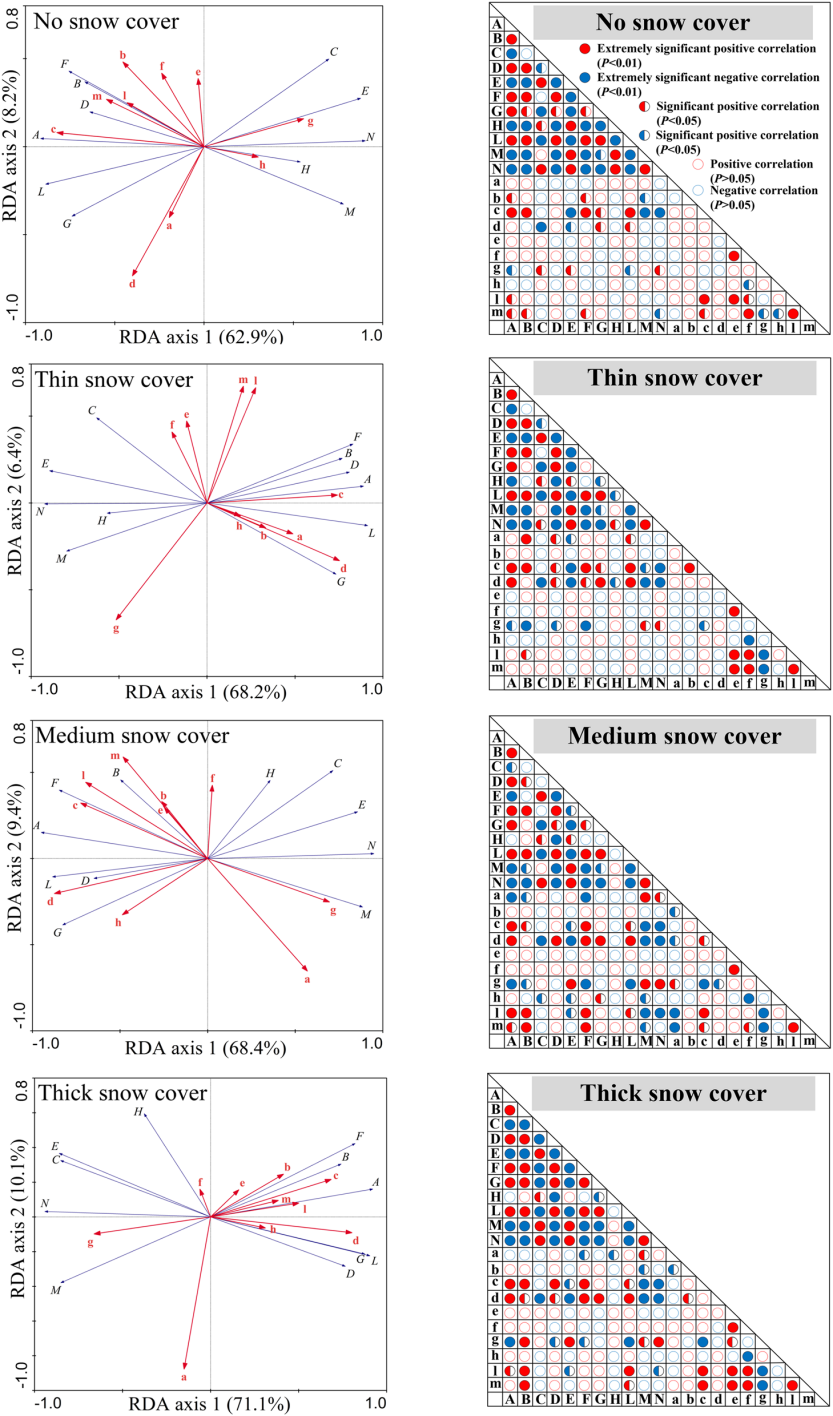
Redundancy analysis (RDA) results and significance level among litter and soil properties. Red solid circles indicate an extremely significant positive correlation (*P* < 0.01); red semicircles indicate a significant positive correlation (*P* < 0.05); red hollow circles indicate a positive correlation (*P* > 0.05); blue solid circles indicate a strong, significant negative correlation (*P* < 0.01); blue semicircles indicate a significant negative correlation (*P* < 0.05); and blue hollow circles indicate a negative correlation (*P* > 0.05). *Note*: A–N represent the litter dry mass, organic carbon, nitrogen, phosphorus, lignin, cellulose, carbon/nitrogen, carbon/phosphorus, carbon/lignin, carbon/cellulose and lignin/cellulose, respectively; a-m represent the soil pH, conductivity, soil water content, bulk density, organic carbon, nitrogen, phosphorus, carbon/nitrogen, carbon/phosphorus and nitrogen/phosphorus, respectively.

The dry mass, C, total P, cellulose and C/lignin of the leaf litter were positively correlated (*P* < 0.05 or *P* < 0.01) with each other, and the dry mass, C and P showed significantly negative correlations with lignin, C/P and C/cellulose (Fig. 6). The dry mass, C, cellulose and C/lignin showed significantly positive correlations with the soil water content, whereas lignin, C/cellulose and lignin/cellulose were negatively correlated (*P* < 0.05) with the soil water content. The N and lignin were negatively correlated (*P* < 0.05) with the soil bulk density. Unlike the correlations under no snow cover, the correlations between dry mass, N, or cellulose and soil water content, or bulk density, the correlations between C/cellulose, or lignin/cellulose and soil bulk density, and the correlations between C, or cellulose and soil P under other snow cover types all reached significant levels (*P* < 0.05 or *P* < 0.01). The number of significant correlations between litter and soil under medium and thick snow cover was greater than that under no and thin snow cover.

## Discussion

### Temporal variations in leaf litter decomposition

The snow cover period (FTP, DEP and TP) showed markedly lower decomposition rates than the snow-free period (PGS, MGS and LGS) in this study. Moreover, the decomposition rate was strongly related to the snow cover condition, land surface temperature, soil water content and litter properties. Snow cover can significantly change the land surface temperature under snow. Thus, the lower rates were probably due to the lower land surface temperatures (− 15–5 °C vs. 5–20 °C) and the condensation of soil water under low temperatures, which might therefore limit the microbial activities and restrict the decomposition of litter during the snow cover period^33–35^.

Significantly higher lignin concentrations were observed in the pregrowing season and middle of the growing season than in the freeze–thaw period and deep-freeze period. It is well known that lignin is more refractory than the majority of other components of litter^36,37^, and the decomposition of lignin was thus relatively slow while the mass of litter decreased rapidly; thus, the lignin concentration in the total litter mass increased. Moreover, the negative correlation between the lignin concentration and soil water content (Fig. 6) further confirmed that high soil water contents can restrict lignin decomposition^38,39^. The continuous freeze–thaw cycle in the snow cover period allowed more snow moisture to move into the mineral soil profile, thereby increasing soil water content and resulting in higher soil water contents than in the snow-free period. Simultaneously, the land surface temperature in the snow-free period (PGS, MGS and LGS) was above 0 ºC, higher than that in the snow cover period (FTP, DEP and TP) (Fig. 2), which therefore elevated the lignin concentration during the latter stages due to the relatively suitable water concentrations and temperatures conducive to the development and colonization of microorganisms and indirectly promoted the accumulation of lignin decomposing microorganisms on the litter^15^. Then, the internal components of the leaves can react with microorganisms to form acid-insoluble components similar to lignin, thus leading to an increase in the proportion of lignin in litter leaves^15^. In contrast to the lignin, cellulose is easily decomposed^36,37^ and can be decomposed and utilized by microbes at the early stage, and lower cellulose concentrations were consequently observed during the freeze–thaw period and deep-freeze period in comparison to during the thaw period, pregrowing season and middle of the growing season. However, frequent freeze–thaw cycles and snowmelt leaching were more likely to cause litter fragmentation, liberating cellulose from litter during the thaw period and therefore increasing the litter cellulose concentration at this stage^15,40^. After the thaw period, the continuous decomposition and utilization of microbial community after the snow cover stages decreased the cellulose concentration, which was largely reduced during the late growing season.

The C concentration was higher in the freeze–thaw period, deep-freeze period and thaw period than in the late growing season. This is because the C concentration is usually positively related to the mass of litter, and the litter C concentration decreased when the mass was reduced, which is in agreement with findings from other studies^17,41,42^. The higher litter N concentration in the snow-free period than the other stages was probably attributed to the relatively higher abundance of microorganisms. The development of a microbial community will cause more exogenous N to be combined into microbial biomass and related by-products^43^. In addition, it may be that the decrease in bulk density was more conducive to the increase in the litter N content (Supplementary Table S2, Figs. 3 and 6). Because reducing the bulk density is more conducive to aeration and water circulation and can increase the migration ability of soil microorganisms, soil microorganisms tend to colonize litter. There have been studies showing that bacteria often have fast growth and turnover rates and contain higher amounts of N and organic compounds^44^. The litter P concentration was higher in the snow cover period than the snow-free period. Because the land surface temperature was below 0 ºC in the snow cover period (Fig. 2), the microbial activities and the decomposition of P were limited. Therefore, the initial concentration was high. In the snow-free period, the P concentration decreased with increasing temperature (Fig. 5). This is in agreement with the finding of Aerts et al.^45^, who showed that an increase in temperature could stimulate P release.

### Effects of snow cover depth on leaf litter decomposition

Our study showed that the mass loss of litter increased with snow cover depth, and a similar finding was reported by Bokhorst et al.^46^, who also found the mass loss rate of litter under heavy snow cover was higher than that under thinner snow cover. This was very likely due to the good thermal insulating ability of snow that could maintain a stable temperature under snow cover^27,47^. The land surface temperature was observed to be 2–4 °C higher and more stable under thick snow cover than that under no or thin snow cover (Fig. 2), which might thereby favor greater litter decomposition rates. The land surface would undergo direct and severe temperature changes if there is lacking seasonal snow protection, which could greatly damage the decomposer community structure and slow the decomposition process^48,49^. In addition, the overall decomposition constant (*k*) in the Schrenk spruce forest (0.294) was lower than that observed in a subtropical forest in Japan (0.37–2.39)^41^, and this difference could be mainly attributed to the longer cold period of the Tianshan Mountains, which spans nearly half of the year. Furthermore, *k* increased from no snow cover to thick snow cover, further indicating that the decomposition rate under heavy snow was relatively fast. These results were therefore consistent with hypothesis two, that is, thick snow would promote the decomposition of litter.

The lignin concentration was higher under thick snow cover than no snow cover in most periods (*P* < 0.05), although the lignin release was opposite. Due to the difference in temperature, thick snow cover provided a relatively stable environment for the microorganisms, which made the microbial community contain more lignin, and the litter lignin concentration increased^25,37,50^. Moreover, lignin was negatively correlated with the soil bulk density (*P* < 0.05), and the correlation was significantly enhanced under thick snow, which may be because the soil C, N, and bulk density under thick snow cover were higher than those under thin snow (Supplementary Table S1). Moreover, this correlation supports hypothesis 3.

The higher cellulose concentration under no snow cover than that under thick snow cover in most stages was likely due to the relatively high microbial activity under thick snow cover, which was more conducive to the decomposition of cellulose, resulting in a decrease of cellulose concentration^26,33,37^. The decomposition of cellulose was also closely related to temperature. Yue et al.^51^ indicated that temperature was an important moderator of cellulose decomposition. In addition, we also found that there were some significant correlations between cellulose and pH, bulk density and soil P under thick snow cover, although there were no such correlations under no snow cover (Fig. 6). This may be related to the physical compaction of thick snow and the litter decomposition rate. These results indicate that the correlation between litter and soil properties may vary with the snow cover depth, which further supports hypothesis 3.

Similar to the litter mass, the discrepancy in litter C concentrations was mainly caused by the difference in the decomposition rates influenced by temperature^46,47^. The litter N concentration was higher under thick snow cover than that under no snow cover. Snow can be a site for atmospheric N deposition. This additional N can be moved into the litterbags as the snow melts, and the thick snow cover area had more snowfall than the other snow cover depths, which would bring more N attached onto the litter surface. Furthermore, the more stable environment under thick snow cover could increase in the mycelium pathway to a certain extent, resulting in an increased litter N concentration^52,53^. The variation in the litter P concentrations under different snow cover depths was mainly due to the litter substrate, the environment, temperature, humidity, and microorganisms^33,34,54^. Microbial activities promoted litter decomposition, thus affecting the release of P, and net fixation affected the microorganisms, i.e., the process of mycelium of fungi, bacteria and actinomycetes transferring P from the soil to the decomposition site of litter. Therefore, there is no uniform law for the change in P between the different snow cover depths. In general, it can be seen from the above discussion that thick snow promotes litter decomposition and accelerates the release of cellulose, C and N to a certain extent, which is similar to our hypotheses two and three.

## Conclusions

In this study, we chose a litterbag field experiment to investigate changes in the characteristics of lignin, cellulose, C, N, and P in litter leaves under seasonal snow cover, including the effects of the decomposition period, snow depth and their interaction on litter decomposition, and we explored the relationship between litter and soil. Previous studies have found that the decomposition coefficient (*k*) of leaf litter increased with snow depth, and *k* ranged from 0.277 to 0.322. The mass loss of leaf litter was enhanced as the snow cover depth increased, and thick snow promoted the decomposition and accelerated the release of cellulose and carbon. The difference in litter decomposition and element release characteristics varier according to the stage. Thicker snow cover promoted the decomposition of litter and accelerated the release of C and nutrients from litter. The snow cover depth changed the relationships between litter properties (e.g., lignin, cellulose and P) and soil properties (e.g., soil water content, bulk density and P); in particular, the relationship among cellulose and soil bulk density and P was strengthened. These results indicated that temporal variations and snow cover depths both affected the entire decomposition process. The findings of this study are helpful for understanding how seasonal snowfall affects forest litter decomposition and surface element turnover in arid areas. In future research, we can further link the plants, litter, and soil to discuss their comprehensive response to seasonal snowfall and ecological trade-offs.

## Supplementary information

Supplementary Information.
