# Supplementary material for: Schrenk spruce leaf litter decomposition varies with snow depth in the Tianshan Mountains
Source: Sci Rep. 2020 Nov 11;10:19556. doi: 10.1038/s41598-020-76368-9 (PMC7658357; doi:10.1038/s41598-020-76368-9)
Supplement: Supplementary file 1 — Supplementary Information. [file 41598_2020_76368_MOESM1_ESM.doc]

**Schrenk spruce leaf litter decomposition varies with snow depth in the Tianshan Mountains**

Lu Gong^1,2*^, Xin Chen^1,2^, Xueni Zhang^1,2^, Xiaodong Yang^1,2^, Yanjiang Cai^3*^

^1^ College of Resources and Environment Science, Xinjiang University, Urumqi 830046, China

^2^ Key Laboratory of Oasis Ecology, Ministry of Education, Urumqi 830046, China

^3^ State Key Laboratory of Subtropical Silviculture, Zhejiang A&F University, Hangzhou 311300, China

**^*^Corresponding author:**

**Lu Gong (L. Gong)**, Email: gonglu721@163.com, College of Resources and Environment Science, Xinjiang University, Urumqi 830046, China

**Yanjiang Cai (Y. Cai)**, Email: yjcai@zafu.edu.cn, State Key Laboratory of Subtropical Silviculture, Zhejiang A&F University, 666 Wusu Street, Lin'an District, Hangzhou 311300, China

Supplementary Materials for “Schrenk spruce leaf litter decomposition varies with snow depth in the Tianshan Mountains”

This MS file includes:

Tables S1 - S3

| **Table S1.** Characteristics of soil properties under different snow cover depth conditions. | | | | |
| --- | --- | --- | --- | --- |
| Property | Snow cover depth | | | |
|  | No | Thin | Medium | Thick |
| pH | 7.38±0.17 a | 7.02±0.17 b | 6.65±0.38 c | 6.56±0.29 c |
| Conductivity (mS/cm) | 0.37±0.10 a | 0.36±0.09 ab | 0.30±0.04 b | 0.33±0.05 ab |
| Soil water content (%) | 25.8±4.43 a | 27.89±5.82 a | 28.11±5.51 a | 29.64±4.56 a |
| Bulk density (g/cm^3^) | 0.85±0.08 a | 0.87±0.09 a | 0.93±0.14 a | 0.92±0.17 a |
| C (g/kg) | 124.93±8.67 b | 125.38±10.21 b | 129.9±8.17 b | 138.9±8.88 a |
| N (g/kg) | 1.19±0.09 b | 1.20±0.12 b | 1.23±0.11 b | 1.33±0.11 a |
| P (g/kg) | 0.49±0.02 b | 0.51±0.04 ab | 0.54±0.06 a | 0.55±0.05 a |

| **Table S2.** Characteristics of soil properties in different decomposition stages. | | | | | | |
| --- | --- | --- | --- | --- | --- | --- |
| Property | Decomposition stage | | | | | |
|  | FTP | DFP | TP | PGS | MGS | LGS |
| pH | 6.64±0.57 b | 6.87±0.38 ab | 6.99±0.4 ab | 7.15±0.28 a | 7.05±0.21 ab | 6.72±0.42 ab |
| Conductivity (mS/cm) | 0.38±0.07 ab | 0.36±0.03 ab | 0.4±0.10 a | 0.22±0.02 c | 0.32±0.02 b | 0.35±0.04 ab |
| Soil water content (%) | 33.15±3.7 a | 28±2.98 b | 33.09±4.16 a | 25.95±3.08 bc | 23.54±2.93 c | 23.41±3.1 c |
| Bulk density (g/cm^3^) | 0.89±0.09 bc | 1.08±0.13 a | 0.98±0.09 b | 0.81±0.03 c | 0.80±0.02 c | 0.80±0.03 c |
| C (g/kg) | 134.97±10.62 a | 125.24±12.55 a | 132.60±11.78 a | 125.25±7.91 a | 128.34±9.18 a | 132.27±7.80 a |
| N (g/kg) | 1.3±0.11 a | 1.17±0.10 a | 1.25±0.14 a | 1.2±0.11 a | 1.24±0.10 a | 1.28±0.12 a |
| P (g/kg) | 0.48±0.04 b | 0.54±0.03 a | 0.48±0.04 b | 0.53±0.05 a | 0.55±0.04 a | 0.56±0.03 a |

| **Table S3**. Mauchly’s test of sphericity for repeated measures ANOVAs of litter properties. | | | | | |
| --- | --- | --- | --- | --- | --- |
| Property | Within-subjects effects | Mauchly's W | χ^2^ | df | Sig. |
| C | Stage | 0.007 | 28.677 | 20 | 0.137 |
| N | Stage | 0.003 | 34.315 | 20 | 0.041 |
| P | Stage | 0.003 | 33.992 | 20 | 0.044 |
| Lignin | Stage | 0.002 | 34.656 | 20 | 0.038 |
| Cellulose | Stage | <0.001 | 46.477 | 20 | 0.002 |
